# Independent Predictors of Hungry Bone Syndrome After Parathyroidectomy for Primary Hyperparathyroidism: Insights from a Large Cohort Study

**DOI:** 10.3390/diagnostics16071041

**Published:** 2026-03-30

**Authors:** Ibrahim Kilinc, Mustafa Oruc, Furkan Savas, Alparslan Ertenlice, Serap Ulusoy

**Affiliations:** 1 Department of General Surgery, Ankara Bilkent City Hospital, Universiteler Mahallesi, Bilkent, Cankaya, 06800 Ankara, Türkiye; 2 Department of General Surgery, Faculty of Medicine, Ankara Yıldırım Beyazıt University, Universiteler Mahallesi, Ihsan Dogramaci Caddesi, Cankaya, 06800 Ankara, Türkiye

**Keywords:** parathyroidectomy, hungry bone syndrome, alkaline phosphatase, postoperative calcium, T-score, thyroid surgery

## Abstract

**Background/Objectives**: Hungry bone syndrome (HBS) is a clinically significant metabolic complication following parathyroidectomy for primary hyperparathyroidism (PHPT), characterized by profound and prolonged hypocalcemia resulting from rapid skeletal remineralization. Although multiple risk factors have been proposed, published data remain inconsistent regarding the most reliable predictors. This study aimed to evaluate potential risk factors associated with the development of HBS and to clarify controversial findings reported in the literature. **Methods**: Patients who underwent surgery for PHPT between January 2019 and May 2025 were retrospectively analyzed. Individuals who developed HBS were compared with those who did not in terms of clinical, biochemical, and surgical parameters. **Results**: HBS occurred in 4.7% of patients. Those in the HBS group exhibited significantly higher preoperative serum calcium, parathyroid hormone, alkaline phosphatase (ALP), and Ca/P ratio, while phosphate, vitamin D, and T-scores were significantly lower. Postoperative day 1 calcium levels were also markedly reduced. Multivariate analysis identified increased ALP, low T-score, concomitant thyroid surgery, and decreased postoperative day 1 calcium as independent predictors. A postoperative day 1 calcium cutoff of 8.6 mg/dL demonstrated strong predictive accuracy. **Conclusions**: HBS is more commonly observed in patients with low bone density and high bone turnover. Patients with these risk factors should be considered high-risk and closely monitored in the early postoperative period to enable prompt intervention and prevent severe complications.

## 1. Introduction

Hungry bone syndrome (HBS) is defined as a condition of prolonged hypocalcemia that develops postoperatively, despite normal parathyroid hormone (PTH) levels, following surgery for primary hyperparathyroidism (PHPT) [1,2,3,4].

However, some studies have included low serum phosphate levels in the definition [5,6]. Others have also incorporated temporal criteria—specifying when hypocalcemia appears and how long it persists postoperatively—into the definition [7,8,9]. Severe and prolonged hypocalcemia in these patients is considered to result from a substantial increase in calcium uptake by bone tissue. This phenomenon is associated with the abrupt cessation of bone resorption following the removal of elevated circulating PTH, while bone formation continues or even accelerates. However, there is no strong evidence directly proving this mechanism [9,10]. In patients without renal failure, increased urinary calcium excretion also contributes to the development of hypocalcemia. In addition, a decrease in PTH-regulated 1,25(OH)_2_D levels may exacerbate the condition by reducing intestinal calcium absorption [11]. In other words, following parathyroidectomy, the PTH stimulus is abruptly removed, causing excessive osteoclastic activity to cease while osteoblastic activity continues for a period. Consequently, calcium uptake by bone increases substantially to support bone formation. This predisposes the patient to symptomatic hypocalcemia, i.e., HBS [12], a complication of parathyroidectomy that adversely affects quality of life, prolongs hospital stays, and increases readmission rates after discharge. The treatment outcomes for the underlying parathyroid disease are thus negatively affected [13,14,15].

Data on the incidence of HBS following parathyroidectomy for PHPT remain limited and inconsistent. Incidence rates reported in the literature range from 8.6% to 59% [1,5,7,8,9,16,17,18]. Various risk factors such as advanced age, elevated preoperative PTH and calcium levels, high bone turnover, skeletal abnormalities, and a rapid decline in serum PTH levels have been reported to contribute to the development of HBS [9,11,19,20,21].

This study aimed to identify potential risk factors for HBS in a large patient cohort and to help clarify the conflicting findings reported in the literature.

## 2. Materials and Methods

### 2.1. Study Design and Population

This retrospective study included all consecutive patients who underwent parathyroidectomy for primary hyperparathyroidism at our institution between January 2019 and May 2025. All operations were performed by attending surgeons from the Department of General Surgery. During this period, 846 patients underwent parathyroidectomy. Seventy-nine patients were excluded due to secondary or tertiary hyperparathyroidism (*n* = 43), familial syndromes such as MEN1 or MEN2A (*n* = 7), incomplete medical records (*n* = 22), or failed parathyroidectomy (*n* = 7). After applying the exclusion criteria, 767 patients were included in the final analysis (Supplementary Figure S1).

### 2.2. Biochemical Measurements

Preoperative, intraoperative, and postoperative biochemical data were obtained from institutional electronic medical records. All biochemical analyses were performed in the central laboratory of our institution using standardized automated analyzers according to the manufacturers’ instructions. Intraoperative PTH monitoring, referred to as “Quick PTH” in this study, was performed using a rapid assay. The clinical rationale for this measurement is to intraoperatively confirm the successful removal of the hyperfunctioning parathyroid tissue; a ≥50% decline in quick PTH levels 10 min after gland excision compared to the pre-incision baseline was accepted as a biochemical cure. Serum albumin, alkaline phosphatase (ALP), calcium, phosphorus, magnesium, blood urea nitrogen (BUN), creatinine, uric acid, estimated glomerular filtration rate (eGFR), and 24 h urinary calcium and phosphorus levels were measured using the Siemens Atellica CH automated chemistry analyzer (Siemens Healthineers, Erlangen, Germany). These biochemical parameters were assessed using IFCC-recommended kinetic colorimetric or enzymatic methods, as appropriate. Parathyroid hormone (PTH) and 25-hydroxyvitamin D [25(OH)D] levels were measured using chemiluminescent immunoassay techniques on the Siemens Atellica IM analyzer, while calcitonin levels were measured using a chemiluminescent immunometric assay on the Siemens IMMULITE analyzer (Siemens Healthineers, Erlangen, Germany). Reference ranges were as follows: albumin 32–48 g/L, ALP 42–98 U/L, calcium 8.7–10.4 mg/dL, phosphorus 2.4–5.1 mg/dL, magnesium 1.3–2.7 mg/dL, BUN 19–49 mg/dL, creatinine 0.5–1.1 mg/dL, uric acid 3.1–7.8 mg/dL, urinary calcium 100–300 mg/24 h, urinary phosphorus 0.4–1.3 g/24 h, and eGFR > 90 mL/min/1.73 m^2^. The reference range for serum PTH was 18.4–80.1 ng/mL. Vitamin D levels < 20 ng/mL were defined as deficient, and levels between 20 and 30 ng/mL were defined as insufficient. Serum calcium concentrations were adjusted for serum albumin levels using the following formula: Corrected Ca (mg/dL) = [0.08 × (40 − albumin [g/L])] + measured Ca level.

### 2.3. Timing of Measurements

Laboratory results were collected at the defined perioperative intervals. Albumin, BUN, uric acid, eGFR, magnesium, and phosphorus values obtained during the final week before surgery were recorded, and the most recent preoperative calcitonin level was used for analysis. PTH assessment included the highest preoperative value obtained within the month preceding surgery, intraoperative measurements, and all postoperative PTH levels measured during the first 6 months of the postoperative period. Similarly, calcium measurements included the highest preoperative value obtained within the month preceding surgery, and all serum calcium values obtained during the first 6 months of the postoperative period were evaluated. For vitamin D, the lowest preoperative value within the month prior to surgery was used. Calcium and phosphorus concentrations from 24 h urine samples collected during diagnostic evaluation were also included.

### 2.4. Imaging and Bone Assessment

Adenoma localization and dimensions were determined based on preoperative ultrasonography reports and operative notes. Parathyroid adenomas were assumed to have an ellipsoid shape, and their volumes were calculated based on ultrasonographic measurements using the formula: volume = (π/6) × length × width × depth. Patients with renal stones detected on ultrasonography or abdominal computed tomography or with a documented history of nephrolithiasis were classified as having a history of renal stones.

Bone health was evaluated using bone mineral density (BMD) values and T-scores obtained from dual-energy X-ray absorptiometry (DXA) of the lumbar spine. Lumbar spine measurements were selected for analysis because they represented the most consistently and widely available DXA data in the retrospective dataset of our institution (lumbar T-scores missing in only 10 patients). Radius, femoral neck, and total hip measurements were not consistently available in our records and were therefore not included in the analysis. Measurements were performed using a GE/Lunar Prodigy densitometer (GE Healthcare, Madison, WI, USA) according to standard acquisition protocols. The precision error for lumbar spine BMD measurements was within acceptable limits, with a coefficient of variation (CV) ≤ 2%, in accordance with international densitometry standards.

### 2.5. Definition of Hungry Bone Syndrome

HBS was defined as a serum albumin-corrected calcium concentration <8.4 mg/dL accompanied by normal or elevated serum PTH levels, occurring on or after postoperative day 3 or persisting for more than 3 days following surgery [22]. Postoperative hypoparathyroidism was defined as hypocalcemia accompanied by low serum PTH levels during the postoperative period. Patients fulfilling this criterion were considered to have postoperative hypoparathyroidism rather than HBS and were excluded from the HBS analysis.

### 2.6. Postoperative Management for Hungry Bone Syndrome

After the diagnosis of HBS, treatment was administered according to the severity of hypocalcemia and the presence of clinical symptoms. All patients with a serum calcium level < 7.0 mg/dL or with severe symptoms of hypocalcemia (including carpopedal spasm, paresthesia, or tetany) received acute intravenous calcium replacement therapy using 10% calcium gluconate. Following clinical and biochemical stabilization, oral supplementation was initiated with a fixed-dose combination containing 1000 mg elemental calcium and 880 IU cholecalciferol (administered as two tablets twice daily) together with calcitriol tablets (0.5 µg twice daily). Patients with serum calcium levels between 7.0 and 8.0 mg/dL or with mild symptoms, such as perioral tingling, were treated with oral supplementation containing 1000 mg elemental calcium and 880 IU cholecalciferol (two tablets once daily) in combination with calcitriol (0.5 µg once daily). Patients with borderline hypocalcemia (serum calcium levels between 8.0 and 8.5 mg/dL) received oral calcium supplementation alone with 1000 mg elemental calcium and 880 IU cholecalciferol (two tablets once daily).

### 2.7. Statistical Analysis

All statistical analyses were performed using R software (version 4.1) (R Foundation for Statistical Computing, Vienna, Austria). Continuous variables are presented as mean ± standard deviation (SD) or median (interquartile range, IQR) according to the data distribution, and categorical variables as frequency (percentage). Normality was assessed using the Shapiro–Wilk test. Comparisons between patients with and without hungry bone syndrome (HBS) were performed using the independent samples *t*-test or the Mann–Whitney U test for continuous variables and the chi-square test or Fisher’s exact test for categorical variables, as appropriate.

Univariate logistic regression analyses were performed to identify potential predictors of HBS. Variables with *p* < 0.10 in the univariate analysis were included in the multivariable logistic regression model to identify the independent predictors. Multicollinearity was assessed using correlation matrices and variance inflation factor analysis. When collinearity was detected, only one correlated variable was retained.

Model calibration was evaluated using the Hosmer–Lemeshow goodness-of-fit test, and the explanatory power was estimated using Nagelkerke R^2^. The area under the receiver operating characteristic (ROC) curve (AUC) was used to assess model discrimination. The optimal classification threshold for the logistic regression model was determined using the Youden index (J = sensitivity + specificity − 1), which identifies the cutoff point on the ROC curve that maximizes the balance between sensitivity and specificity. Given the low prevalence of HBS (4.7%), model performance was additionally evaluated at a high-specificity operating point to assess the model’s ability to rule in HBS with high confidence. Sensitivity, specificity, positive predictive value (PPV), and negative predictive value (NPV) are reported at both thresholds. Internal validation was performed using three complementary approaches. First, 10-fold stratified cross-validation was conducted to estimate out-of-sample discriminative performance. Second, bootstrap resampling with 200 iterations was used to estimate the optimism (difference between apparent and test performance) and compute the optimism-corrected AUC. Third, leave-one-out cross-validation (LOOCV) was performed as an additional assessment of model stability. Model calibration was further evaluated using the Brier score. The significance of regression coefficients was assessed using the Wald test. All statistical tests were two-tailed, and a *p*-value of <0.05 was considered statistically significant.

## 3. Results

### 3.1. Patient and Lesion Characteristics

A total of 767 patients were included, with a mean age of 53 ± 12 years, and the majority were women (620; 80.8%). Among them, 36 (4.7%) developed HBS. A history of renal stones was present in 169 (23.9%) patients. Parathyroid carcinoma was detected in 5 patients (0.7%) in the cohort, and concomitant thyroid surgery was performed in 251 (32.7%) patients. The median parathyroid gland volume was significantly larger in patients with HBS (1765.1 mm^3^ [IQR 402.5–3367.7] vs. 345.5 mm^3^ [158.4–816.8], *p* < 0.001). In addition, concomitant thyroid procedures were significantly more common in the HBS group (lobectomy, 12 (33.3%); total thyroidectomy, 14 (38.9%); vs. 117 (16.0%) and 108 (14.8%), respectively; *p* < 0.001). Parathyroid carcinoma occurred in 4 (11.1%) patients with HBS, compared with 1 (0.1%) non-HBS patient (*p* < 0.001) (Table 1).

### 3.2. Preoperative Laboratory Findings

Patients who developed HBS demonstrated more severe biochemical abnormalities before surgery. Serum total calcium levels were significantly higher in the HBS group (12.2 vs. 11.4 mg/dL, *p* < 0.001), while phosphorus levels were lower (2.11 vs. 2.66 mg/dL, *p* < 0.001), resulting in an elevated calcium-to-phosphorus ratio (6.91 vs. 4.33, *p* < 0.001). Preoperative parathyroid hormone (PTH) levels were markedly higher in patients with HBS (877 vs. 189 pg/mL, *p* < 0.001), and alkaline phosphatase (ALP) levels were also significantly increased (289 vs. 106 U/L, *p* < 0.001). Vitamin D concentrations were lower in the HBS group (9.7 vs. 16.0 ng/mL, *p* < 0.001). Bone mineral density was reduced in patients with HBS (0.85 vs. 0.99 g/cm^3^, *p* < 0.001), with a lower T-score (−2.6 vs. −1.4, *p* < 0.001). Additionally, 24 h urinary calcium excretion was significantly higher among those who developed HBS (460 vs. 323 mg/day, *p* < 0.001) (Table 2).

### 3.3. Postoperative Laboratory Findings

Patients who developed HBS also exhibited more significant postoperative biochemical abnormalities. On postoperative day 1, serum calcium levels were significantly lower in the HBS group (7.74 vs. 8.88 mg/dL, *p* < 0.001). Similarly, serum magnesium levels were reduced (1.69 vs. 1.81 mg/dL, *p* < 0.005). Postoperative phosphorus levels were also lower in patients with HBS. At the six-month follow-up, serum calcium remained slightly lower in the HBS group (9.17 vs. 9.46 mg/dL, *p* < 0.001), and PTH levels were modestly higher (60 vs. 45 pg/mL, *p* < 0.05). The percentage reduction in PTH from pre- to postoperative measurements was significantly greater in HBS patients than in non-HBS patients (97.3% vs. 90.9%, *p* < 0.001) (Table 3).

### 3.4. Predictors of HBS

Univariate analysis identified several clinical and biochemical variables associated with HBS development. Significant predictors included low preoperative phosphorus levels, high preoperative ALP levels, high PTH levels, high Ca/P ratio, low vitamin D levels, presence of bone disease, low T-score, large parathyroid gland volume, and concomitant thyroid surgery (all *p* < 0.05). Among the postoperative parameters, low calcium, magnesium, and phosphorus levels, greater postoperative PTH reduction, and high intraoperative quick PTH were also significantly associated with HBS (all *p* < 0.05). To facilitate clinical interpretation, odds ratios for continuous variables with small per-unit effects are reported per clinically meaningful increments: ALP per 10 U/L, PTH per 100 pg/mL, parathyroid volume per 1000 mm^3^, and urinary calcium per 100 mg/day (Table 4). Because preoperative ALP and PTH levels demonstrated collinearity, only ALP was retained in the multivariable model (Pearson r = 0.694). Four variables were included in the final multivariable logistic regression analysis: preoperative ALP, T-score, concomitant thyroid surgery, and postoperative calcium levels.

In multivariable analysis, preoperative ALP (OR = 1.161 per 10 U/L, 95% CI 1.105–1.219, *p* < 0.001), concomitant thyroid surgery (OR = 2.998, 95% CI 1.030–8.722, *p* = 0.044), T-score (OR = 0.514 per 1 SD unit, 95% CI 0.330–0.801, *p* = 0.003), and postoperative calcium (OR = 0.369 per 1 mg/dL, 95% CI 0.208–0.653, *p* < 0.001) were identified as independent predictors of HBS (Table 5). These results indicate that for every 10 U/L increase in preoperative ALP, the odds of developing HBS increase by 16.1%, whereas each 1 mg/dL increase in postoperative calcium is associated with a 63.1% reduction in the odds of developing HBS.

**Figure 1:**
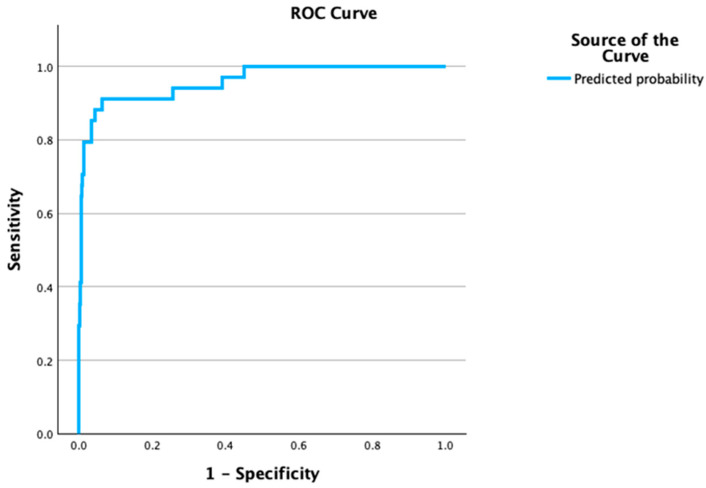
Receiver operating characteristic (ROC) curve for the multivariable logistic regression model predicting hungry bone syndrome (HBS) after parathyroidectomy. The model demonstrated excellent discrimination, with an area under the curve (AUC) of 0.923 (*p* < 0.001), sensitivity of 58.8%, specificity of 99.3%, positive predictive value (PPV) of 80%, and negative predictive value (NPV) of 98.1%.

The model demonstrated excellent discrimination, with an AUC of 0.92 (*p* < 0.001). The Hosmer–Lemeshow goodness-of-fit test indicated adequate calibration (*p* = 0.417), and the Nagelkerke R^2^ was 0.619. At the high-specificity operating point, the model yielded a sensitivity of 58.8%, specificity of 99.3%, positive predictive value (PPV) of 80%, and negative predictive value (NPV) of 98.1% (Figure 1). At the Youden-optimal threshold, the sensitivity increased to 88.2% with a specificity of 95.8%, demonstrating the model’s ability to capture the majority of HBS cases when a balanced classification threshold is applied.

Internal validation confirmed the stability of the model with minimal overfitting. Bootstrap resampling (200 iterations) estimated an optimism of 0.012, yielding an optimism-corrected AUC of 0.91. The ten-fold stratified cross-validation yielded a concordant AUC of 0.95, and the leave-one-out cross-validation yielded an AUC of 0.94. The Brier score was 0.021, further supporting good model calibration.

**Figure 2:**
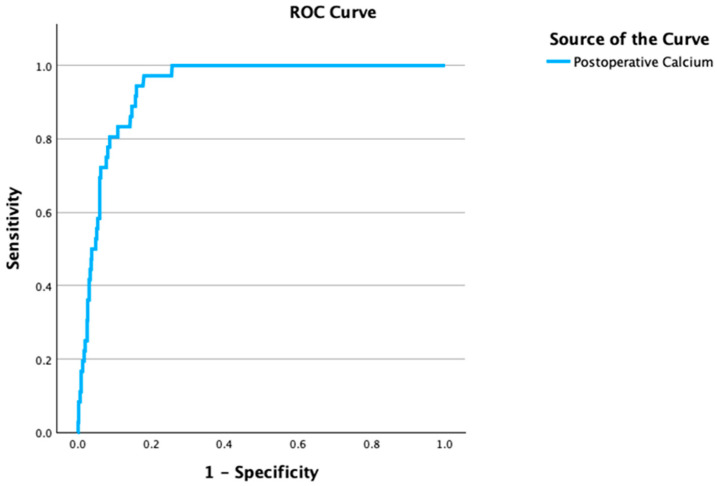
Receiver operating characteristic (ROC) curve for postoperative calcium levels in predicting hungry bone syndrome (HBS) after parathyroidectomy. A postoperative calcium threshold of 8.62 mg/dL demonstrated excellent discriminative ability with an area under the curve (AUC) of 0.93 (*p* < 0.001), sensitivity of 90%, and specificity of 82%.

A cutoff analysis for postoperative calcium identified a threshold of 8.62 mg/dL, yielding an AUC of 0.93 (*p* < 0.001) with 90% sensitivity and 82% specificity. Postoperative calcium levels were included as a continuous variable in the model to maximize statistical power (Figure 2).

### 3.5. Postoperative Course and Treatment Outcomes

In patients diagnosed with hungry bone syndrome (HBS), hypocalcemia developed at a mean of 2.4 days postoperatively. Following the diagnosis of HBS, calcium and calcitriol replacement therapy was initiated, and subsequent dose adjustments were made according to the patients’ clinical status and serial serum calcium measurements. Treatment completion was defined as the time point at which serum calcium levels returned to the normal range without the need for calcium or calcitriol supplementation. Based on this definition, the mean duration of replacement therapy was 23 days. All patients recovered with replacement therapy except for one. In this case, which was excluded from the calculation of the mean treatment duration, supplementation continued for six months; however, no follow-up data were available beyond this period.

## 4. Discussion

This study investigated the clinical, biochemical, and surgical factors associated with HBS in patients with PHPT. Preoperative ALP levels, T-scores, and undergoing concomitant thyroid surgery were identified as independent predictors of HBS development. In addition, low postoperative day 1 calcium levels were associated with HBS and may serve as an early postoperative marker for identifying patients at increased risk. In this study, HBS developed in 4.7% of patients, which is relatively lower than the rates reported in some previous studies [2]. The definition of hungry bone syndrome varies across the literature [5,6,7,8,9], with differences in calcium thresholds, duration of hypocalcemia, and additional biochemical parameters used for diagnosis. In this study, we adopted a previously reported definition describing HBS as albumin-corrected calcium <8.4 mg/dL accompanied by normal or elevated PTH levels occurring on or after postoperative day 3 or persisting for more than 3 days. This approach was chosen to distinguish HBS from transient postoperative hypocalcemia and hypocalcemia related to postoperative hypoparathyroidism. Differences in diagnostic criteria across studies may partly explain the wide variation in the reported incidence of HBS. The relatively low incidence observed in our cohort may therefore partly reflect the relatively strict diagnostic criteria applied in this study. These findings emphasize the central role of bone turnover in HBS pathogenesis, as reflected by elevated preoperative alkaline phosphatase levels and low bone mineral density. In addition to skeletal metabolism, concomitant thyroid surgery may contribute to this risk, suggesting that combined procedures can exacerbate postoperative calcium fluctuations. Moreover, early postoperative calcium levels serve as an immediate and reliable marker for the onset of HBS.

Serum ALP levels are regarded as a biochemical marker of bone remineralization [23], and by reflecting the preoperative bone turnover rate, they provide indirect information about the extent of osteoclastic activity and bone resorption [9]. The identification of ALP as a predictive factor for HBS in our study aligns with the findings of several previous reports that demonstrate an association between ALP levels and the development of HBS [11,16,18]. However, our study is the first to identify the T-score, a measure of bone mineral density, as an independent predictor of HBS. Accordingly, patients with a low T-score, indicating reduced bone mineral density, are at an increased risk of HBS due to increased postoperative calcium flux into bone tissue. In this study, we also found that the incidence of HBS was significantly higher in patients with PHPT and osteopenia or osteoporosis, clinically reflecting the T-score. Accordingly, our results suggest that preoperative low T-scores and elevated ALP levels are two complementary parameters that can be assessed together for the early prediction of HBS.

For the postoperative day 1 serum calcium level, which we identified as an additional predictor in our study, a cutoff value of 8.6 mg/dL was established. Guillén Martínez et al. [18] also reported that postoperative day 1 and week 1 serum calcium levels were significantly lower in patients who developed HBS. These data support that an early postoperative decrease in calcium is a significant marker for the development of HBS. The 8.6 mg/dL cutoff value identified in our series aligns with the findings of their study and offers a clinically meaningful threshold for predicting HBS risk in the early postoperative period. The postoperative day 1 calcium level in patients undergoing parathyroidectomy for PHPT may therefore facilitate early identification of individuals at high risk of HBS and prompt initiation of appropriate replacement therapy.

The surgical factor identified as a predictor in our study was whether patients underwent concomitant thyroid surgery. Some studies have reported a higher frequency of HBS in patients who underwent thyroid surgery concomitant with parathyroidectomy [1,18,24]. The increased occurrence of HBS in patients undergoing concomitant thyroid surgery has been attributed to the indirect effects of thyroid surgery on the parathyroid glands [18]. It has been suggested that hypoparathyroidism, resulting from impaired vascularization or trauma to the parathyroid glands during surgery, may contribute to the development of HBS. However, all patients who developed hypoparathyroidism were excluded from our analysis. Additional mechanisms, which could be explored in future studies, may thus also contribute to the higher incidence of HBS observed in patients undergoing concomitant thyroid surgery.

Although not identified as predictors in this study, several variables that remain controversial in the literature were also found to be significantly associated with HBS. Among these factors, preoperative PTH levels and pathological gland volume were highly correlated with serum ALP levels, and the ALP level was thus included in the multivariate analysis model. Based on these findings, preoperative PTH levels and increased pathological gland volume can be considered clinically relevant markers associated with HBS. This finding aligns with those of previous studies reporting an association of preoperative PTH levels [1,8,22] and pathological gland size [5,18] with HBS. Moreover, previous studies have reported associations between preoperative calcium [5,25] and phosphate levels [25,26], which are influenced by PTH secretion, and the development of HBS as well as severe hypocalcemia. Consistent with this, this study also found an association between preoperative calcium levels, phosphate levels, the calcium/phosphate ratio, and the development of HBS.

Another contentious issue in the literature is the relationship between HBS and vitamin D levels. Salman et al. [27] reported lower vitamin D levels in patients with HBS, whereas Chandran et al. [22] did not detect any significant difference. In this study, consistent with the findings of Salman et al., vitamin D levels were significantly lower in patients who developed HBS. Among postoperative parameters, when comparing patients with and without HBS, significant differences in magnesium, calcium, and PTH levels at 6 months were also observed. Based on these postoperative findings, the importance of monitoring patients’ magnesium levels can be emphasized. Full recovery from HBS may exceed 6 months, and follow-up periods should therefore be sufficiently long.

Evaluation of the parathyroid gland’s pathological findings revealed a significant difference in the distribution of pathological diagnoses between patients who developed HBS and those who did not. In the HBS patient group, the proportion of adenomas was lower, whereas the proportions of carcinomas and atypical tumors were higher. This finding may be attributed to relatively higher preoperative hormone secretion in cases of carcinoma and atypical tumors [28,29,30], resulting in greater postoperative bone remineralization.

Regarding model performance, the modest sensitivity (58.8%) at the high-specificity threshold reflects the expected trade-off in a low-prevalence setting, prioritizing the identification of very high-risk patients for aggressive prophylaxis. Conversely, the Youden-optimal threshold demonstrated high sensitivity (88.2%), making it more appropriate for general screening. Importantly, the consistently high negative predictive value (98.1%) and robust internal validation confirm the model’s clinical utility in safely ruling out HBS.

This study’s findings may aid in identifying high-risk patients in clinical practice. Specifically, in patients identified as at-risk based on the predictors discussed, calcium replacement can be initiated in the early postoperative period before hypocalcemia develops. In patients identified as high-risk for HBS, preventive strategies may be considered, including optimization of vitamin D status before surgery, closer postoperative biochemical monitoring, and early initiation of calcium and calcitriol supplementation. Such measures may help reduce the severity of postoperative hypocalcemia and facilitate earlier recognition and management of HBS.

Regarding limitations, despite its retrospective design, our study includes a larger patient population and more HBS cases than found in the literature. Our study extends the existing knowledge on HBS and provides a foundation for developing more effective strategies for its prediction, prevention, and management. Furthermore, the predictive factors identified in this study could be used to develop a scoring system for HBS in the future. This study is also limited by the absence of external validation. Although internal validation using bootstrap resampling, 10-fold cross-validation, and leave-one-out cross-validation demonstrated minimal overfitting (optimism = 0.012), prospective validation in an independent cohort is warranted. The events-per-variable ratio (9:1) approaches but does not exceed the commonly recommended threshold of 10:1, which may limit the stability of coefficient estimates; however, the model remained robust across all internal validation approaches. An additional limitation is that bone mineral density (BMD) assessment was restricted to the lumbar spine; the unavailability of cortical bone data may have precluded the full demonstration of the T-score’s potential predictive power. Another limitation of this study relates to its retrospective design. Detailed information regarding subsequent dose adjustments, additional supplementation requirements, and the cumulative doses of calcium and calcitriol during follow-up was not consistently available in the medical records. The treatment regimens described in this study therefore reflect the initial management of HBS rather than the complete course of dose titration during the follow-up period.

## 5. Conclusions

Among patients undergoing surgery for primary hyperparathyroidism, preoperative ALP levels, postoperative calcium levels, T-scores, and the presence of concomitant thyroid surgery were identified as independent predictors of HBS. In addition, a cutoff value of 8.6 mg/dL was established for the postoperative day 1 calcium level. Integrating these markers into clinical practice can facilitate early implementation of preventative measures against HBS.

## Figures and Tables

**Table 1:** Comparison of patient and lesion characteristics between patients with and without HBS.

Variables	Total (*n* = 767)	HBS (−) (*n* = 731)	HBS (+) (*n* = 36)	*p*-Value
Age, years, mean, SD	53 ± 12	53 ± 12	50 ± 14	0.356
Sex, male, *n*, %	147 (19.2)	140 (19.2)	7 (19.4)	0.965
History of Renal Stones, *n*, %	169 (22.7)	161 (22.7)	8 (22.2)	0.946
Parathyroid volume mm^3^, median, IQR	357.8 (159.8–918.7)	345.5 (158.4–816.8)	1765.1 (402.5–3367.7)	**<0.001**
Parathyroid Location, *n*, %				0.253
Left Superior	89 (11.7)	86 (11.8)	3 (8.3)	
Left Inferior	276 (36.2)	268 (36.9)	8 (22.2)	
Right Inferior	251 (32.9)	237 (32.6)	14 (38.9)	
Right Superior	91 (11.9)	85 (11.7)	6 (16.7)	
Intrathyroidal	37 (4.9)	33 (4.5)	4 (11.1)	
Atypic	18 (2.4)	17 (2.3)	1 (2.8)	
Parathyroid Pathology *n*, %				**<0.001**
Adenoma	702 (90.9)	674 (91.5)	28 (77.8)	
Atypic adenoma	27 (3.5)	24 (3.3)	3 (8.3)	
Hyperplasia	32 (4.2)	31 (4.2)	1 (2.8)	
Cancer	5 (0.7)	1 (0.1)	4 (11.1)	
Concomitant Thyroid Surgery, *n*, %				**<0.001**
No	516 (67.3)	506 (69.2)	10 (27.8)	
Lobectomy	129 (16.8)	117 (16)	12 (33.3)	
Total Thyroidectomy	122 (15.9)	108 (14.8)	14 (38.9)	
Thyroid Pathology, *n*, %				0.551
Benign	146 (58.1)	132 (58.7)	14 (53.8)	
Malignant	105 (41.9)	93 (41.3)	12 (46.4)	

Significant values (*p* < 0.05) are highlighted in bold.

**Table 2:** Comparison of preoperative laboratory findings between patients with and without HBS.

Variables	Total (*n* = 767)	HBS (−) (*n* = 731)	HBS (+) (*n* = 36)	*p*-Value
Calcium, mg/dL, median, IQR	11.5 (11.1–12)	11.4 (11.1–11.9)	12.2 (11.5–13.2)	**<0.001**
Phosphorus, mg/dL, mean, SD	2.63 ± 0.56	2.66 ± 0.54	2.11 ± 0.21	**<0.001**
Magnesium, mg/dL, median IQR	2.06 (1.91–2.19)	2.06 (1.9–2.18)	2.08 (2–2.24)	0.134
Ca/P, ratio, median, IQR	4.36 (3.75–5.12)	4.33 (3.72–5.02)	6.91 (4.53–8.22)	**<0.001**
Albumin, g/L, Mean ± SD	38.95 ± 3.34	38.93 ± 3.34	39.29 ± 3.42	0.312
Creatinine, mg/dL, median, IQR	0.73 (0.64–0.85)	0.74 (0.64–0.85)	0.72 (0.64–0.93)	0.774
GFR, mL/min/1.73 m^2^, median, IQR	98.3 (85.1–106.8)	98.11 (85–106.6)	101.5 (87.9–116.9)	0.112
BUN, mg/dL, median, IQR	29 (23.5–34.2)	29.2 (23.54–34.2)	25.64 (21.6–38.4)	0.277
Uric Acid, mg/dL, median, IQR	5.1 (4.3–5.9)	5.1 (4.3–5.9)	5.1 (4.35–5.75)	0.964
PTH, pg/mL, median, IQR	192 (139–273)	189 (137–264)	877 (265–1387.5)	**<0.001**
Calcitonin, pg/mL, median, IQR	2 (2–2)	2 (2–2)	2 (2–2.04)	0.799
Quick PTH, pg/mL, median, IQR	34 (23–65)	33 (23–61)	81 (53–159)	**<0.001**
Vitamin D, ng/mL, median, IQR	15.56 (10.4–22.1)	16(10.8–22.4)	9.7 (8.1–15.2)	**<0.001**
ALP, U/L, median, IQR	108 (85–137)	106 (84–131)	289 (190–559)	**<0.001**
Urine calcium, mg/day, median, IQR	326 (241–452)	323 (240–441)	460 (346–524)	**<0.001**
Urine Phosphorus, mg/day, median, IQR	0.78 (0.56–0.93)	0.78 (0.56–0.93)	0.84 (0.53–1.1)	0.69
BMD g/cm^3^, median, IQR	0.99 (0.87–1.1)	0.99 (0.88–1.1)	0.85 (0.75–0.97)	**<0.001**
T Score	−1.4 (−2.4–−0.5)	−1.4 (−2.4–−0.5)	−2.6 (−3.2–−1.8)	**<0.001**

Significant values (*p* < 0.05) are highlighted in bold.

**Table 3:** Comparison of postoperative laboratory findings between patients with and without HBS.

Variables	Total (*n* = 767)	HBS (−) (*n* = 731)	HBS (+) (*n* = 36)	*p*-Value
Calcium POD * 1, mg/dL, median, IQR	8.85 (8.42–9.32)	8.88 (8.49–9.36)	7.74 (7.43–7.99)	**<0.001**
Magnesium, mg/dL, median, IQR	1.81 (1.66–1.98)	1.81 (1.67–1.98)	1.69 (1.54–1.85)	**<0.005**
Phosphorus, mg/dL, mean, SD	3.25 ± 0.68	3.28 ± 0.64	2.56 ± 0.91	**<0.005**
Calcium 6 months, mg/dL, median, IQR	9.46 (9.13–9.78)	9.46 (9.17–9.79)	9.17 (8.73–9.5)	**<0.001**
PTH, pg/mL, median, IQR	16 (8–35)	16 (8–35)	25 (10.5–35)	0.395
6 months PTH, pg/mL, median, IQR	45 (32–65)	45 (32–64)	60 (34–92.5)	**<0.05**
PTH reduction ratio, median, IQR	91.16 (81.18–96.12)	90.9 (80.6–95.9)	97.33 (91.4–98.6)	**<0.001**

Significant values (*p* < 0.05) are highlighted in bold. * POD: Postoperative Day.

**Table 4:** Univariate logistic regression analysis of risk factors for HBS.

Variables	Univariate OR	95% CI	*p*-Value
Preoperative Calcium	1.007	0.977–1.037	0.656
Preoperative Phosphorus	0.093	0.046–0.189	**<0.001**
Preoperative Ca/P Ratio	1.262	1.044–1.525	**<0.05**
Preoperative PTH *	1.690	1.480–1.929	**<0.001**
Quick PTH *	1.128	1.073–1.187	**<0.001**
Vitamin D	0.936	0.889–0.985	**<0.05**
Preoperative ALP *	1.189	1.134–1.246	**<0.001**
Urinary Calcium *	1.278	1.097–1.489	**<0.005**
Bone Mineral Density	1.001	1.000–1.003	**0.138**
T-Score	0.499	0.369–0.674	**<0.001**
Presence of Bone Disease	3.207	1.314–7.828	**<0.05**
Parathyroid Volume *	1.210	1.072–1.365	**<0.005**
Presence of Thyroid Surgery	2.513	1.710–3.691	**<0.001**
Postoperative Calcium	0.142	0.083–0.242	**<0.001**
Calcium < 8.36 mg/dL	154.3	21.3–1115.9	**<0.001**
Postoperative Magnesium	0.197	0.063–0.622	**<0.01**
Postoperative Phosphorus	0.199	0.118–0.337	**<0.001**
Postoperative PTH Reduction Ratio	1.099	1.036–1.167	**<0.005**

Abbreviations: OR, odds ratio; CI, confidence interval; PTH, parathyroid hormone; ALP, alkaline phosphatase; * Odds ratios are reported per clinically meaningful increments: PTH per 100 pg/mL, Quick PTH per 10 pg/mL, ALP per 10 U/L, urinary calcium per 100 mg/day, and parathyroid volume per 1000 mm^3^. All other continuous variables are reported per 1-unit increase. Significant values (*p* < 0.05) are highlighted in bold.

**Table 5:** Multivariate logistic regression analysis of independent predictors for HBS.

Variables	Wald	Multivariate OR	95% CI	*p*-Value
Preoperative ALP (per 10 U/L)	29.7	1.161	1.105–1.219	**<0.001**
T-Score (per 1 SD unit)	8.65	0.514	0.330–0.801	**0.003**
Concomitant Thyroid Surgery	4.03	2.998	1.030–8.722	**0.044**
Postoperative Calcium (per 1 mg/dL)	11.69	0.369	0.208–0.653	**<0.001**

Significant values (*p* < 0.05) are shown in bold. Hosmer and Lemeshow: *p* = 0.417|Nagelkerke R^2^ = 0.619|AUC = 0.92 Optimism-corrected AUC = 0.91|Brier score = 0.021.

## Data Availability

The data presented in this study are available on request from the corresponding author. The data are not publicly available due to privacy and ethical restrictions.

## Supplementary Materials

The following supporting information can be downloaded at: https://www.mdpi.com/article/10.3390/diagnostics16071041/s1, Figure S1: Patient selection flowchart.
