# Supplementary material for: Independent Predictors of Hungry Bone Syndrome After Parathyroidectomy for Primary Hyperparathyroidism: Insights from a Large Cohort Study
Source: Diagnostics (Basel). 2026 Mar 30;16(7):1041. doi: 10.3390/diagnostics16071041 (PMC13073297; doi:10.3390/diagnostics16071041)

Patient selection flowchart

Independent predictors of hungry bone syndrome after parathyroidectomy

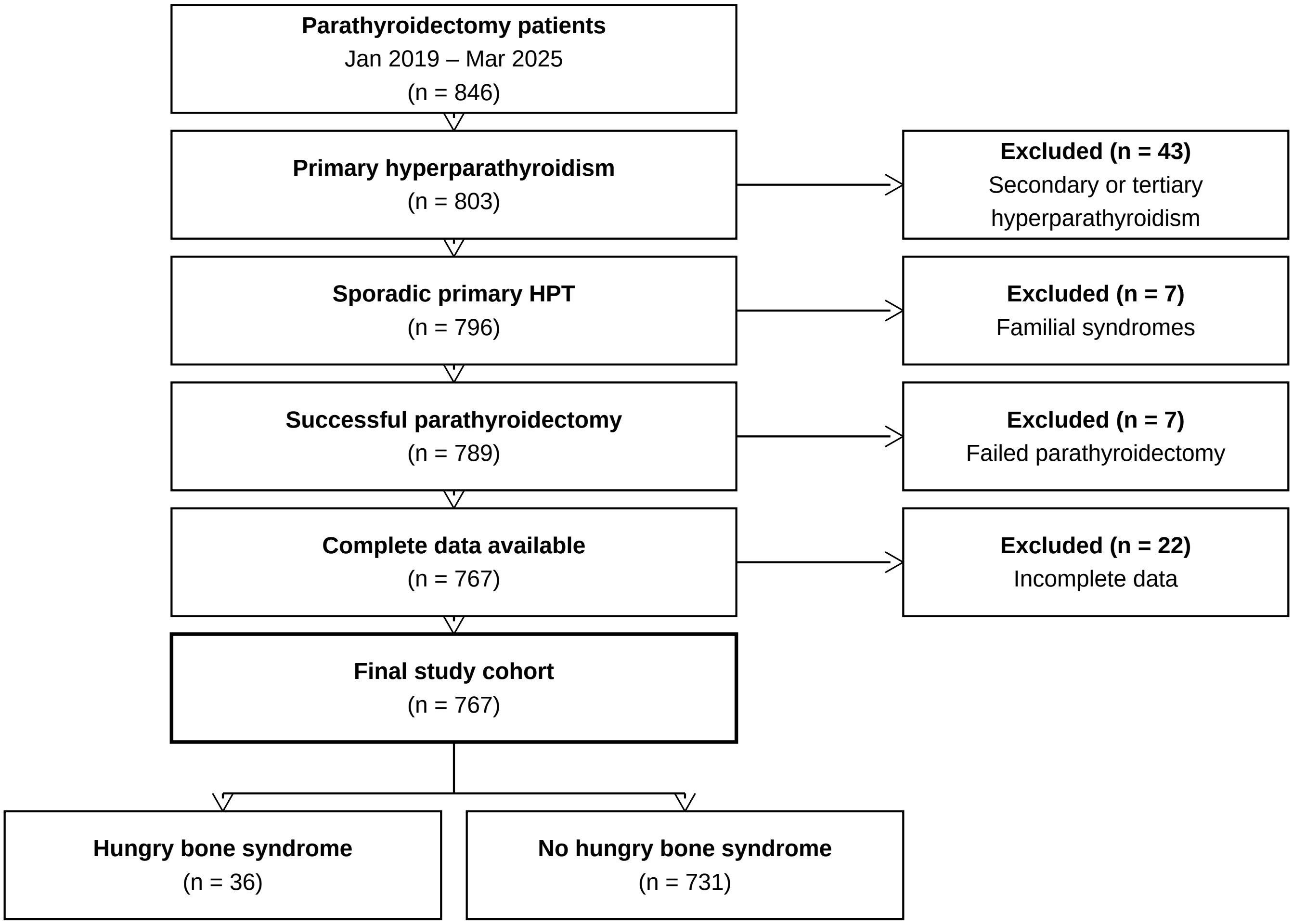

Supplement: Supplementary file 1 [file diagnostics-16-01041-s001.zip › diagnostics-4209062-supplementary.pdf]
